# Limited medial osteochondral lesions of the talus associated with chronic ankle instability do not impact the results of endoscopic modified Broström ligament repair

**DOI:** 10.1186/s13018-022-02968-y

**Published:** 2022-02-03

**Authors:** Shi-Ming Feng, Jie Chen, Chao Ma, Filippo Migliorini, Francesco Oliva, Nicola Maffulli

**Affiliations:** 1 Orthopaedic Department, Sports Medicine Department, Xuzhou Central Hospital, Xuzhou Clinical College of Xuzhou Medical University, No. 199, the Jiefang South Road, Xuzhou, 221009 Jiangsu People’s Republic of China; 2 Department of Orthopaedic, Trauma, and Reconstructive Surgery, RWTH University Hospital, Pauwelsstraße 30, 52074 Aachen, Germany; 3 Department of Musculoskeletal Disorders, Faculty of Medicine and Surgery, University of Salerno, Salerno, Italy; 4 Guy Hilton Research Centre, School of Pharmacy and Bioengineering, Keele University, Stoke-on-Trent, Staffordshire ST4 7QB England; 5 Centre for Sports and Exercise Medicine, Barts and The London School of Medicine and Dentistry, Mile End Hospital, 275 Bancroft Road, London, E1 4DG England

**Keywords:** Osteochondral lesion, Chronic lateral ankle instability, Broström–Gould procedure, Arthroscopic microfracture

## Abstract

**Background:**

The arthroscopic modified Broström procedure, with repair of the anterior talofibular ligament and extensor retinaculum, produces good functional outcomes in patients with chronic lateral ankle instability (CLAI). CLAI can be associated with osteochondral lesions of the talus (OLTs). It remains unclear whether associated limited OLTs affect clinical outcomes in such patients.

**Methods:**

This retrospective cohort study included 92 CLAI patients with and without OLTs undergoing an all-inside arthroscopic modified Broström procedure from June 2016 to May 2019. The patients were divided into non-lesion group (*n* = 32) and lesion group (*n* = 60) according to whether CLAI was associated or not with OLTs. All the osteochondral lesions less than 15 mm in diameter were managed with bone marrow stimulation techniques (arthroscopic microfracture) at the time of the arthroscopic modified Broström procedure. The Visual Analogue Scale (VAS) scores, American Orthopedic Foot and Ankle Society (AOFAS) scores, Karlsson Ankle Function Score (KAFS), Anterior Talar Translation (ATT), Active Joint Position Sense (AJPS), and the rate of return to sports were compared in both groups.

**Results:**

Increase in all the functional scores (VAS, AOFAS, KAFS, ATT, and AJPS) in both groups was, respectively, recorded 1 year and 2 years after surgery. At the 1-year and 2-year follow-up, there was no significant difference in the VAS, AOFAS, KAFS, ATT, and AJPS scores between the non-lesion and lesion groups.

**Conclusion:**

In patients with CLAI who underwent an arthroscopic modified Broström procedure, the presence of limited OLTs (less than 15 mm in diameter), which required arthroscopic microfracture, did not exert any influence on outcome.

**Level of Evidence:**

Level III, a retrospective comparative study.

## Background

**Fig. 1:**
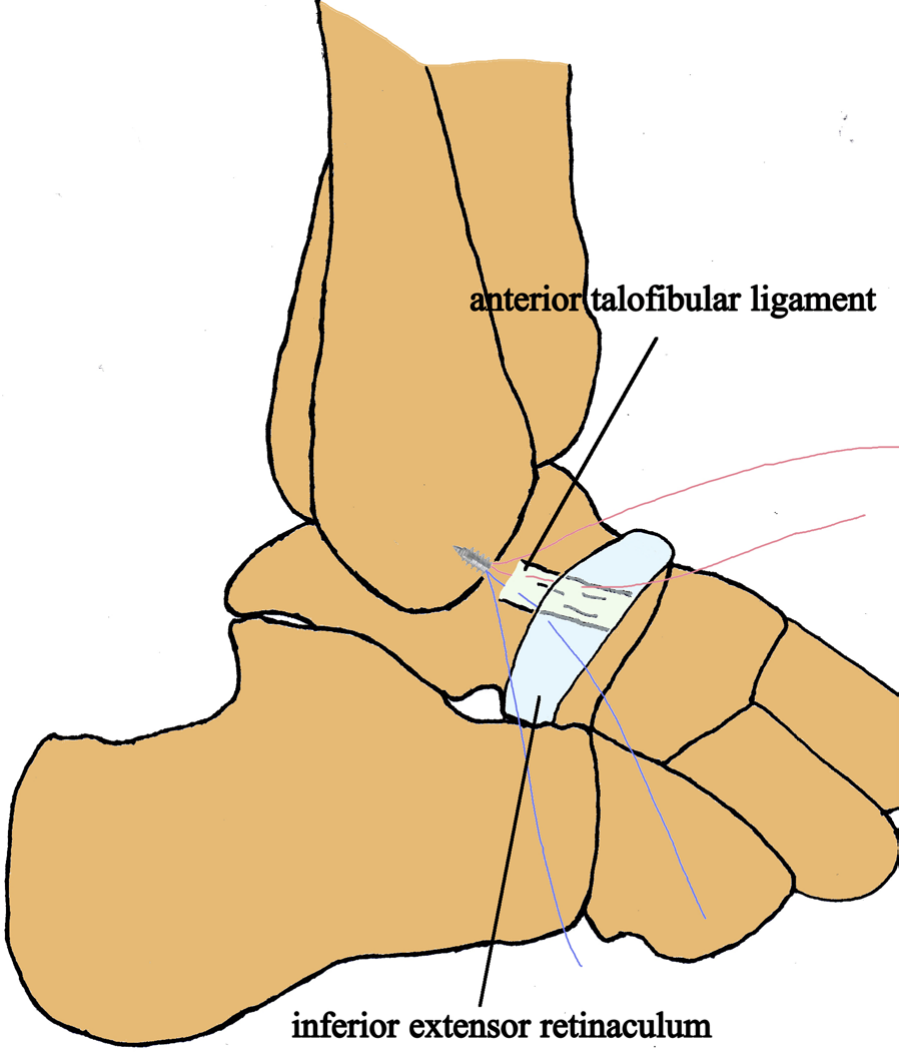
The diagram of the modified Broström procedure

Chronic lateral ankle instability (CLAI) can be associated with osteochondral lesions of the talus (OLTs), which occur in up to 70% of acute ankle sprains and associated fractures [1]. More than 17% CLAI patients suffered osteochondral lesions [2, 3]. The modified Broström procedure (repair of the anterior talofibular ligament and augmentation with the inferior extensor retinaculum, Fig. 1) is a standard procedure for CLAI patients who do not respond to three to six months of conservative management [4–6]. Patients with OLTs less than 15 mm in diameter are usually treated with intra-articular debridement and microfracture [1, 7]. In patients with CLAI and OLTs, lateral ankle stabilization and simultaneous surgery for the intra-articular ailment are indicated [8–10].

In CLAI patients, associated osteochondral lesions are typically located medially in the coronary plane and centrally in the sagittal plane [11]. Compared with isolated osteochondral lesions, osteochondral lesions in CLAI are larger, and additional chondral lesions at the tip of the medial malleolus are seen [12]. OLTs might lead to persistent pain and osteoarthritis if not managed properly [13]. Arthroscopic repair of the lateral ankle ligamentous complex and management of intra-articular lesions of the ankle are now routinely performed [14]. However, it is unclear whether the presence of OLTs affects the outcome of the stabilization procedure.

The purpose of this study was to compare the two-year clinical results in terms of ankle function and stability in patients with isolated CLAI in whom the arthroscopic modified Broström procedure had been performed, and in patients with CLAI associated with OLTs in whom intra-articular debridement and bone marrow stimulation (microfracture) had been performed in association with the arthroscopic modified Broström procedure. We hypothesized that the arthroscopic microfracture for OLTs treatment might have some negative effects on the clinical outcomes of CLAI.

## Patients and methods

The investigation was a retrospective cohort study evaluating the clinical outcomes of an arthroscopic modified Broström procedure with or without microfracture in CLAI patients with or without OLTs. After obtaining institutional review board approval (ID: XZXY-LJ-20151210-108, Xuzhou Central Hospital), prospectively collected data of CLAI patients underwent arthroscopic modified Broström procedure between June 2016 to May 2019 were retrospectively reviewed. All patients provided a signed informed consent as well as consent under the Health Insurance Portability and Accountability Act to participate in this study.

### Patient selection

Inclusion criteria were as follows: (1) CLAI patients with no improved symptoms after 6 months of conservative management; (2) patients who had undergone unilateral ankle arthroscopic modified Broström procedure with one double-loaded suture anchor fixation (Fastin RC 3.5 mm, Smith & Nephew, Andover, MA); (3) a preoperative magnetic resonance imaging of the ankle showed medial OLTs or no OLTs; (4) OLTs located in the medial portion of the talus, with a diameter no greater than 15 mm in diameter and a depth no greater than 8 mm; (5) follow-up for at least 24 months with complete surgical and follow-up data; (6) all procedures were performed by the same senior foot and ankle surgeon with extensive experience in arthroscopy, who was not involved in postoperative follow-up.

Participants were excluded if they had any of the following: (1) multiple OLTs; (2) OLTs requiring procedures other than microfracture; (3) ankle osteoarthritis or multiple ligament injuries; (4) MRI evidence of injury to the medial ligamentous complex of the ankle or clinical evidence of medial ankle instability; (5) previous history of foot and ankle surgery or secondary injuries to the ankle.

### Participants

**Fig. 2:**
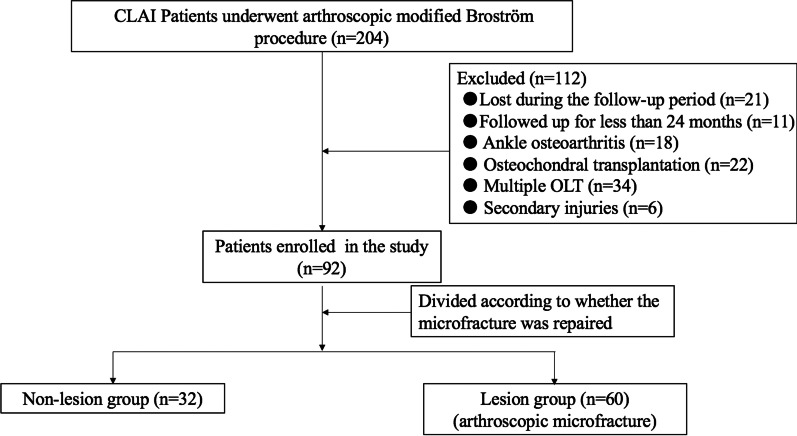
The flow diagram of the study. CLAI, chronic lateral ankle instability; OLT, osteochondral lesion of the talus

From June 2016 to May 2019, 204 consecutive CLAI patients underwent an arthroscopic modified Broström procedure performed by a senior fellowship trained foot and ankle surgeon. Twenty-one patients were lost to follow-up, 11 were followed up for less than 24 months, 18 had ankle osteoarthritis, 22 were treated with osteochondral transplantation, in 34 CLAI was associated with multiple OLTs, and 6 had secondary injuries. Eventually, a total of 92 CLAI patients satisfied the above inclusion and exclusion criteria and are reported in the present investigation (Fig. 2).

Depending on whether an arthroscopic microfracture procedure had been performed, the patients were divided into two groups. In the non-lesion group (*n* = 32), the anterior talofibular ligament and the capsule were sutured, and the extensor retinaculum was transferred to strengthen the lateral ankle ligament in the modified Broström procedure. In the lesion group (*n* = 60), arthroscopic microfracture of the talus and the modified Broström procedure (the same as in the non-lesion group) were performed. Patients of the lesion group were classified into two subgroups (stage 2 group with 32 patients and stage 3 group with 28 patients) according to the Hepple classification system [15].

The two groups (lesion group vs. non-lesion group) and the two subgroups (stage 2 group vs. stage 3 group) were comparable in terms of age, body mass index, preoperative visual analogue score (VAS), American Orthopaedic Foot and Ankle Society (AOFAS) score, Karlsson Ankle Functional Score (KAFS), Anterior Talar Translation (ATT), and other general conditions (Tables 1 and 2).

**Table 1:** Characterization of the sample

Variable	Lesion group (*n* = 60)	Non-lesion group (*n* = 32)	*P*^a^ value
Age, y	33.48 ± 8.37	35.28 ± 9.88	0.360^b^
Sex			0.827^c^
Male	38	21	
Female	22	11	
BMI, kg/m^2^	24.71 ± 2.78	24.03 ± 2.71	0.266^b^
VAS	6.08 ± 1.64	6.25 ± 2.00	0.668^b^
AOFAS	69.72 ± 9.29	71.79 ± 9.70	0.278^b^
KAFS	66.42 ± 8.58	67.91 ± 11.36	0.519^b^
ATT, mm	7.42 ± 1.80	7.22 ± 1.31	0.584^b^
Disease duration, mo	16.13 ± 3.23	15.63 ± 2.92	0.459^b^

BMI, Body mass index; OCD, osteochondral defect; VAS, Visual Analogue Scale; AOFAS, American Orthopaedic Foot and Ankle Society; KAFS, Karlsson Ankle Function Score; ATT, Anterior Talar Translation

^a^A value *p* < 0.05 was set as statistically significant

^b^*t* test

^c^Pearson *χ*^2^ test

**Table 2:** Comparison of the Hepple stage 2 and stage 3 patients of the lesion group

Variable	Hepple stage 2 group (*n* = 32)	Hepple stage 3 group (*n* = 28)	*P*^a^ value
Age	32.38 ± 8.88	34.75 ± 7.73	0.277^b^
BMI	24.62 ± 2.49	24.81 ± 3.13	0.792^b^
VAS	6.03 ± 2.01	6.14 ± 1.11	0.795^b^
AOFAS	70.22 ± 9.15	69.14 ± 9.57	0.658^b^
KAFS	66.03 ± 8.11	66.86 ± 9.23	0.713^b^
ATT	7.41 ± 1.78	7.43 ± 1.85	0.962^b^
Disease duration, mo	15.72 ± 3.24	16.61 ± 3.21	0.291^b^

BMI, Body mass index; VAS, Visual Analogue Scale; AOFAS, American Orthopaedic Foot and Ankle Society; KAFS, Karlsson Ankle Function Score; ATT, Anterior Talar Translation

^a^A value *p* < 0.05 was set as statistically significant

^b^*t* test

### Surgical technique

With the patient supine, a 7-cm pillow was placed under the ipsilateral hip after induction of anesthesia. The affected leg was placed over the distal edge of the operating table, and a pneumatic tourniquet placed on the thigh was inflated to 300 mmHg after exsanguination of the limb.

Firstly, all the patients underwent arthroscopic evaluation and treatment before the ligament repair through standard anterolateral and anteromedial portals. For patients with OLTs, after debridement of the articular cartilage lesion and subchondral cyst, the OLT was carefully measured, and the microfracture was performed to promote revascularization of the lesion.

Subsequently, an accessory portal at the anterior margin of the tip of the fibula was established to examine the lateral ligament complex, and expose and freshen the anterior talofibular ligament footprint on the fibula. A double-loaded suture anchor (Fastin RC 3.5 mm, Smith & Nephew, Andover, MA) was inserted into the distal fibula at the mid-portion of the footprint region. The anterior talofibular ligament and the inferior extensor retinaculum were sutured as previously described [16]. With the operated ankle placed at 5° of eversion, the suture knot was tightened with a knot pusher.

### Postoperative management

Early non-weight-bearing functional exercises and isometric exercises of the injured limb were performed after the procedure under the assistance of a rehabilitation specialist. With mild dorsiflexion and eversion, a short leg brace was used to immobilize the operated ankle joint for 2 weeks. An Aircast™ (DJO, Vista, CA, USA) boot was applied for the next 4 weeks. After 6 weeks, the patients were allowed to gradually resume normal physical activity.

### Evaluation of outcomes

All the measurements were taken at 1 year and 2 years after surgery by an experienced foot and ankle surgeon who was unaware of the procedure performed. The varus stress test of the bilateral ankle joint, the anterior drawer test, and ATT score were evaluated to assess ankle stability. The VAS, AOFAS, KAFS, and return to sports were evaluated to assess ankle function. The Active Joint Position Sense (AJPS) using the active joint angle reproduction test was evaluated to assess ankle proprioception [17]. The patients were seated on a height-adjustable table with the affected foot placed at a 90° angle from the hip, knee, and ankle. The affected ankle was passively placed in 10° and 20° of inversion and plantar flexion using the footplate, respectively. Patients were then asked to actively place the foot in these positions. Every patient was tested three times, and the average was used for statistical purposes.

### Statistical analysis

SPSS version 17.0 software (SPSS, Inc., Chicago, IL, USA) was used to analyze the data. The quantitative variables were expressed as mean ± standard deviation. The measurement data (VAS, AOFAS, KAFS, ATT, and AJPS scores) in each group before and after surgery, as well as between two groups after surgery, were compared using the Student’s *t* test (for normal distribution) or the Mann–Whitney test (for asymmetric distribution). The Pearson Chi-square test was used to compare categorical variables. A post hoc power analysis was performed. Differences of *p* < 0.05 were considered statistically significant.

## Results

All operations in the two groups were successful, and the incisions healed primarily without complications. Microfracture of the talus was performed in all the patients in the OLT group. The hospital duration between the two groups was similar. In the non-lesion group, shorter operative time compared to the lesion group was evident. The varus stress test was negative, and Anterior Talar Translation was negative (grade 0) in both groups. The mean time to the return to normal activity for patients in the lesion group and the non-lesion group was comparable (Table 2). All the functional scores in both groups (AOFAS, KAFS, and AJPS) were significantly improved at 1 year and 2 years after surgery. In both groups, results were comparable for VAS, AOFAS, KAFS, ATT, and AJPS at 1 year and 2 years (Table 2). Group sample sizes of 60 and 32 achieve less than 20.00% power (AOFAS, KAFS, ATT and AJPS, respectively) to reject the null hypothesis of equal means, with a significance level (alpha) of 0.050 using a two-sided two-sample unequal-variance t-test (Table 3). Subgroup analysis (Table 4) showed that no difference was identified in the evaluation items before and after surgery in different stages of the OLTs except for plantar flexion 20° score. In the OLT group, patients with Hepple stage 2 lesions experienced greater plantar flexion 20° scores in the AJPS test compared to Hepple stage 3 patients at 2 years after surgery (Table 4).

**Table 3:** Functional outcomes comparison of the two groups

Variable	Lesion group (*n* = 60)	Non-lesion group (*n* = 32)	*P*^a^ value	Power^d^
Operative time, min	49.45 ± 6.57	45.16 ± 9.91	0.015^b^	
Hospital duration, day	3.55 ± 0.59	3.56 ± 0.67	0.927 ^b^	
VAS				
1 year	0.00 (0.00–1.00)	0.00 (0.00–2.00)	0.902^c^	
2 years	0.00 (0.00–1.00)	0.00 (0.00–1.00)	0.861^c^	
AOFAS				
1 year	90.75 ± 4.63	91.50 ± 3.64	0.429^b^	0.135
2 years	91.67 ± 4.46	92.13 ± 3.80	0.623^b^	0.081
KAFS				
1 year	90.13 ± 4.22	89.38 ± 4.52	0.425^b^	0.119
2 years	91.12 ± 4.26	90.19 ± 4.12	0.316^b^	0.171
ATT, mm				
1 year	2.48 ± 1.10	2.53 ± 0.80	0.828^b^	0.057
2 years	2.80 ± 1.15	2.69 ± 0.93	0.613^b^	0.078
AJPS, degree				
1 year				
Inversion 10°	7.70 ± 1.36	7.47 ± 1.08	0.407^b^	0.141
Inversion 20°	17.70 ± 1.59	18.06 ± 1.56	0.297^b^	0.178
Plantar flexion 10°	7.62 ± 1.15	7.78 ± 1.16	0.516^b^	0.095
Plantar flexion 20°	18.35 ± 1.29	18.19 ± 1.42	0.580^b^	0.082
2 years				
Inversion 10°	8.00 ± 1.51	8.13 ± 1.10	0.651^b^	0.075
Inversion 20°	17.95 ± 1.64	18.28 ± 1.55	0.350^b^	0.156
Plantar flexion 10°	7.83 ± 1.24	8.06 ± 1.34	0.414^b^	0.124
Plantar flexion 20°	18.50 ± 1.41	18.59 ± 0.98	0.738^b^	0.064

VAS, Visual Analogue Scale; AOFAS, American Orthopaedic Foot and Ankle Society; KAFS, Karlsson Ankle Function Score; ATT, Anterior Talar Translation; AJPS, Active Joint Position Sense

^a^A value *p* < 0.05 was set as statistically significant

^b^*t* test

^c^Mann–Whitney test

^d^Power is computed to reject the null hypothesis of equal means

**Table 4:** Functional outcomes comparison of the Hepple stage 2 and stage 3 patients of the lesion group

Variable			Hepple stage 2 group (*n* = 32)	Hepple stage 3 group (*n* = 28)	*P*^a^Value
Operative time, min			50.16 ± 5.89	48.64 ± 7.29	0.378^b^
Hospital duration, day			3.63 ± 0.61	3.46 ± 0.58	0.300^b^
VAS	1 yr		0.0 (0.0–.0)	0.0 (0.0–2.0)	0.173^c^
	2 yr		0.0 (0.0–1.0)	0.0 (0.0–1.0)	0.225^c^
AOFAS	1 yr		89.88 ± 4.20	91.75 ± 4.96	0.421^b^
	2 yr		91.31 ± 4.69	92.07 ± 4.23	0.390^b^
KAFS	1 yr		89.72 ± 4.39	90.61 ± 4.05	0.119^b^
	2 yr		91.56 ± 4.12	90.61 ± 4.43	0.515^b^
ATT	1 yr		2.34 ± 1.18	2.64 ± 0.99	0.296^b^
	2 yr		2.81 ± 1.23	279 ± 1.07	0.929^b^
AJPS	1 yr	Inversion 10°	7.41 ± 1.27	8.04 ± 1.40	0.073^b^
	1 yr	Inversion 20°	17.59 ± 1.76	17.82 ± 1.39	0.584^b^
	1 yr	Plantar flexion 10°	7.78 ± 1.13	7.43 ± 1.17	0.240^b^
	1 yr	Plantar flexion 20°	18.34 ± 1.18	18.36 ± 1.42	0.968^b^
	2 yr	Inversion 10°	7.97 ± 1.68	8.04 ± 1.32	0.865^b^
	2 yr	Inversion 20°	17.94 ± 1.65	17.96 ± 1.67	0.950^b^
	2 yr	Plantar flexion 10°	8.03 ± 1.20	7.61 ± 1.26	0.188^b^
	2 yr	Plantar flexion 20°	18.97 ± 1.12	17.96 ± 1.53	0.006^b^

VAS, Visual Analogue Scale; AOFAS, American Orthopaedic Foot and Ankle Society; KAFS, Karlsson Ankle Function Score; ATT, Anterior Talar Translation; AJPS, Active Joint Position Sense

^a^A value *p* < 0.05 was set as statistically significant

^b^*t* test

^c^Mann–Whitney test

At the final follow-up, in the non-lesion group, 27 patients had resumed the pre-injury sports activities and 5 patients were involved in leisure sports activities because of fear of secondary injury to the surgery site; in the OLT group, 52 patients had returned to pre-injury sports activities and 8 patients chose non-intense exercise because of fear of secondary injury to the surgery site. The exercise participation rate was similar between the two groups (*p* = 0.763).

## Discussion

The most important finding of the present study was that CLAI patients with or without limited medial OLTs achieved comparable functional outcomes following the arthroscopic modified Broström procedure and, if necessary, microfractures. No significant differences were found postoperatively between the lesion group and the non-lesion group in VAS, AOFAS, KAFS, ATT, AJPS, time of return to normal activity, or the rate of return to pre-injury sports.

The arthroscopic modified Broström procedure, with anterior talofibular ligament and inferior extensor retinaculum suture, is an established treatment of CLAI [16, 18–20]. Arthroscopic joint debridement and microfracture are indicated for the management of limited (no more than 15 mm in diameter and 8 mm in depth) OLTs [21, 22]. OLTs are commonly encountered in CLAI patients undergoing surgery and are mostly located over the centromedial portion and the anterolateral portion of the talus [23, 24]. It is more likely that OLTs of the medial portion of the talus are present in patients with long-standing CLAI [25] and rotational instability of the ankle [26]. In CLAI patients, internal–external rotation, anterior–posterior translation, and inversion in flexion increase: the medial side of the talus experiences greater sagittal plane motion within the mortise, resulting in increased shear forces and overload along this area [27, 28]. Thus, OLTs on the medial talar dome are more commonly associated with CLAI as a result of rotational instability of the ankle [29]. In 33 CLAI patients with OLTs who underwent arthroscopic modified Broström procedure, OLT patients experienced poorer surgical outcomes [30]. Ikoma et al. [26] found that CLAI patients with OLT had smaller talus lesions and lower stages (stage 1 and stage 2) than OLT patients without CLAI. The lower grades (1 and 2) of OLTs were associated with better surgical outcomes than the higher grades (3 and 4), and the smaller lesions were associated with better clinical outcomes [10].

Debridement, microfracture, osteochondral autografts, or autologous chondrocyte transplantation have all been used in symptomatic OLT patients [31–36]. Bone marrow stimulation, performed using arthroscopic microfracture, is indicated if full-thickness defects and unstable articular cartilage lesions of limited area and depth are observed [37]. Arthroscopic microfracture provided excellent and reliable outcomes for OLTs with a diameter no greater than 15 mm regardless of the location of the OLT [38]. Corr et al. [39] retrospectively analyzed 45 consecutive patients with OLTs treated using arthroscopic microfracture. After a minimum follow-up of 10 years, 93.3% survival rate and 85.7% return to sport were observed. Legnani and colleagues [40] retrospectively analyzed and compared the outcomes of 76 CLAI cases (capsular shrinkage group: 42 ankles; OLT group: 34 ankles) treated with an arthroscopic modified Broström procedure and microfracture. At a median follow-up of 6 years, significant improvements of the VAS, AOFAS, and Tegner scores were recorded in both groups. The arthroscopic modified Broström procedure with microfracture should be considered a reasonable method for CLAI patients with OLTs [41]. The modified Broström procedure and microfracture are a viable surgical option for the patient with CLAI and OLTs [42, 43]. In fact, the size of the OLT in the patients involved in the present study was limited (no more than 15 mm in diameter): the presence of an OLT was not associated with poorer functional results.

Returning to pre-injury sports activities is a major goal of surgery [43, 44]. In the present study, the VAS and AOFAS scores were comparable. The VAS and AOFAS scoring systems focus mostly on the subjective evaluation of joint pain: OLTs do not affect negatively postoperative outcome. The KAFS and AJPS mainly focus on objective assessment of ankle function and were similar in the two groups, indicating that the arthroscopic modified Broström procedure resulted in excellent ankle stability and comparable function for CLAI patients with and without OLTs. Based on the above results, it appears that the rate of return to sport is not related to the presence of an OLT in CLAI patients.

This major strengths of the present study are that stability, ankle function, and proprioception of the operated ankles were carefully assessed by fully trained surgeons. Also, the study was adequately powered, and a relatively large sample of patients was included. Nevertheless, this study has some limitations. First, only the AJPS was used for proprioceptive function assessment. Although every position was repeated 3 times, the method might not comprehensively assess all aspects of the proprioception of the affected ankle. Second, the present investigation was a retrospective study, with inevitable potential patient selection bias. However, all the data were collected prospectively, and all measurement procedures were performed in a strict scientific fashion. It would be difficult to perform a randomized controlled trial in patients in whom CLAI is associated with OLTs. Obviously, it would be ethically untenable to perform in such patients only a lateral ligamentous complex stabilization without addressing the OLTs. Third, the duration of follow-up was 2 years. The reparative fibrocartilage after microfracture is mechanically inferior to hyaline cartilage and deteriorates over time: it is still unclear whether these favorable clinical outcomes will remain stable over time. However, as the experience in the use of arthroscopic modified Broström surgery continues to grow, these issues will be undoubtedly addressed.

## Conclusions

Limited medial osteochondral lesions of the talus associated with chronic ankle instability do not impact negatively the results of endoscopic modified Broström ligament repair, isolated arthroscopic modified Broström procedure, as shown by AOFAS, KAFS, ATT, AJPS, and exercise participation rate. It is still unclear whether the all-inside arthroscopic Broström procedures offer similar functional outcomes to the traditional open surgery or whether the other location of OLTs may exert any influence on clinical results. Future studies are needed to clarify these issues.

## Data Availability

The datasets generated during and/or analyzed during the current study are available throughout the manuscript.

## Abbreviations

CLAI: Chronic lateral ankle instability
OLTs: Osteochondral lesions of the talus
VAS: Visual Analogue Scale
AOFAS: American Orthopedic Foot and Ankle Society scores
KAFS: Karlsson Ankle Function Score
ATT: Anterior Talar Translation
AJPS: Active Joint Position Sense
